# Oral mite anaphylaxis (pancake syndrome) caused by storage mite Acarus siro and its treatment with allergen immunotherapy 

**DOI:** 10.5414/ALX02415E

**Published:** 2023-04-28

**Authors:** Tanjana Preiser-Funke, Karl-Christian Bergmann

**Affiliations:** 1 Nuss-Anaphylaxie Netzwerk e.V., Wiesbaden, and; 2 Institute of Allergology, Charité – Universitätsmedizin Berlin, corporate member of Freie Universität Berlin and Humboldt-Universität zu Berlin, Berlin, Germany, and Fraunhofer Institute for Translational Medicine and Pharmacology ITMP, Allergology and Immunology, Berlin, Germany

**Keywords:** idiopathic anaphylaxis, food allergy, mites

## Abstract

A 10-year-old atopic patient with asthma, peanut, and house dust mite allergy developed frequent episodes with symptoms including abdominal pain, nausea, vomiting, dizziness, drop of blood pressure, and occasionally shortness of breath and wheezing. After detailed diagnostics including an ISAC test and several other specific IgE blood tests, which could not explain the symptoms, the patient tested positive for specific IgE to *Acarus siro* (flour mites) (9.2 kU/L). As no oral food challenge with *Acarus siro* was available, the patient’s family implemented avoidance measures by storing food containing flour in the refrigerator, and the patient started subcutaneous immunotherapy (SCIT) with Depigoid Acarus siro. The implementation of avoidance measures led to an immediate improvement of symptoms, and after 3 years of treatment, products containing flour, stored at room temperature, are tolerated again.

## Background 

House dust and storage mites are known as inhalation allergens, which may cause asthma and allergic rhinitis [1]. 

Oral mite anaphylaxis – also known as pancake syndrome – is considered a rare disease, which has been mainly reported in tropical and subtropical countries [2, 3, 4, 5] but also in northern countries [6]. 

Dust and storage mites are also present in Germany, so possible reactions to ingestion should not be neglected [7]. But oral mite anaphylaxis is rare and not well known by clinicians, so it is likely to be overlooked. 

*Acarus siro* can be part of sealed food packages, so complete avoidance is impossible. However, diagnosed patients can contribute to reduce the amount of contamination by mite allergens by avoiding long storage periods and by storing food in the refrigerator to stop mite proliferation. 

In addition, subcutaneous immunotherapy (SCIT) is a well-established treatment option for house dust mite allergies [8]. For storage mites, there are only named patient products available. It is likely, but not yet proven, that these treatment options could be effective for allergies caused by ingestion. 

## Case presentation 

Over the course of 2 years, a 10-year-old atopic patient, living in the south of Germany, with allergic asthma, environmental allergies (house dust mite, cat, horse, dog), and a peanut allergy, developed recurring episodes with potentially allergic symptoms including abdominal pain, nausea, vomiting, dizziness, drop of blood pressure, and occasionally shortness of breath and wheezing, especially after consumption of shortly heated products containing flour, like pancake, semolina pudding, or waffles. The severity and frequency of symptoms increased over time. Interestingly, the symptoms were significantly reduced during vacation periods abroad but persisted or even worsened during vacation periods at home. Due to the high frequency and intensity of reactions, the patient was not able to attend school on a regular basis anymore, had to spend most of the time in bed, and lost weight. The patient started preferring food consisting of fruits, because he had the impression that this did not cause symptoms. 

A very strong reaction with shortness of breath, wheezing, and drop of blood pressure to 70/50 mmHg took place after tasting pollen directly from a beehive. Other family members who also tasted the pollen tolerated it. 

### Examinations 

Detailed specific IgE testing for suspected foods and pollen, including an ISAC test, was performed which could not explain the reactions, including negative results for specific IgE for tropomyosin rAni s 3, nBla g 7, rDer p 10, nPen m 1. 

Non-allergic reasons were also excluded, for example a brain tumor, cystic fibrosis, celiac disease, or Crohn’s disease. 

Specific IgE for mites was tested with several positive results (Table 1). 

According to a fact sheet of the University of Michigan, five Acarus species have been recorded from nests of European honeybee (*Apis mellifera*): *Acarus farris, Acarus gracilis, Acarus immobilis, Acarus siro,* and *Acarus tyrophagoides *[9]. 

*Acarus siro* lives in wheat flour and also in pollen stored in beehives (pollen is rich in protein and serves as nutrition for the bee larvae but is also suitable as food for storage mites). So *Acarus siro* could be the common cause of all reactions. 

### Treatment 

The family implemented measures to avoid mites. This immediately led to a significant reduction of symptom frequency and severity and an improvement of the boy’s quality of live. 

Additionally, a treatment with SCIT using Depigoid Milben-Mix (LETI Pharma GmbH, Ismaning, Germany) for house dust mites (January 2019 until May 2022) and Depigoid Acarus siro (April 2019 until July 2022) was performed, which led to further improvements. 

### Outcome 

After the implementation of avoidance measures, the patient was able to attend school on a regular basis. After 6 months of treatment with SCIT, the patient started to do sports again, and after 3 years of treatment, the patient has completely recovered. As of today, the patient is free of anaphylactic symptoms, even without avoidance measures for mites. The asthma persists. 

## Discussion 

Unfortunately, no samples of foods which caused reactions are available to verify whether *Acarus siro* was contained. The patient lives in a rural area with a piggery and a chicken coop in the neighborhood, so there are potential sources for an *Acarus siro* population available. 

The avoidance measures for mites had a strong effect, and the parents had not informed the patient beforehand about those measures to avoid placebo effects. Nevertheless, a placebo-by-proxy effect or simple coincidence is not likely but possible. 

The SCITs for house dust mites and *Acarus siro* were done in parallel. Therefore, it cannot be distinguished which treatment had an effect. A placebo effect would be possible, too. 

## Practical considerations 

The prevalence of mite-induced food allergies might be significantly underestimated. It has a strong impact on patient’s health. 

Therefore, consider house dust and storage mites as potential cause for allergic reactions to food that cannot be explained by a food allergy. 

After detection of specific IgE for mites, instruct patients to avoid long storage of products containing flour at room temperature. 

Consider allergen immunotherapy if avoidance measures improve the symptoms. 

## Informed consent 

We confirm that we have received informed consent from the patient’s caregivers for this case report. 

## Funding 

We have not received any funding for this article. 

## Conflict of interest 

We, the authors, confirm that we have no conflict of interest. 

**Table 1.** Summary: Allergen-specific IgE results (December 12, 2018; total IgE 1,092 kU/L).

**Allergen**	**Value (kU/L)**
*Acarus siro*	9.2
*Euroglyphus maynei*	29.1
*Tyrophagus putreus*	2.0
*Blomia tropicalis*	0.5
*Lepidoglyphus destructor*	< 0.3
*Dermatophagoides farinae*	> 100
*Dermatophagoides pteronyssinus*	> 100

Other laboratory results: blood eosinophils 250/ul (October 20, 2021).
